# Swept Source Optical Coherence Tomography Analysis of a Selected Eye’s Anterior Segment Parameters in Patients with Pseudoexfoliation Syndrome

**DOI:** 10.3390/jcm11010268

**Published:** 2022-01-05

**Authors:** Michał Dembski, Anna Nowińska, Klaudia Ulfik-Dembska, Edward Wylęgała

**Affiliations:** 1 Chair and Clinical Department of Ophthalmology, Faculty of Medical Sciences in Zabrze, Medical University of Silesia in Katowice, 40-055 Katowice, Poland; anna.nowinska@sum.edu.pl (A.N.); klaudia.ulfik@gmail.com (K.U.-D.); rekroz@sum.edu.pl (E.W.); 2 Ophthalmology Department, Railway Hospital in Katowice, 40-760 Katowice, Poland

**Keywords:** cornea, keratometry, pseudoexfoliation syndrome, optical coherence tomography, open-angle glaucoma, anterior chamber angle

## Abstract

Background: Pseudoexfoliation syndrome (PEX) is a systemic, age-related disorder characterized by the production and accumulation of pseudoexfoliation material. To date, detailed characteristics have not been published regarding the iridocorneal angle and cornea in patients with pseudoexfoliation syndrome determined through swept source optical coherence tomography (SS-OCT). Methods: A total of 150 eyes of patients with pseudoexfoliation syndrome (ages 69–86 years) and 166 eyes in a control group (ages 54–79 years) were analyzed. Results: The central corneal thickness in the PEX group was 549.56 μm and was slightly (insignificantly) higher than that of the control group (540.56 µm). The anterior chamber of the eye was visibly shallower in patients with PEX syndrome than in those of the control group (2.49 mm vs. 3.07 mm; *p* < 0.001). The Fourier analysis parameters of the cornea showed multiple differences between the PEX and control groups. With respect to iris area, the parameters showed statistically significant differences between the PEX and control groups in all four quadrants of the eye. No statistical significance was found in the PEX group for the iridocorneal angle parameters, or corneal and lens parameters depending on gender and age. Conclusions: PEX syndrome is characterized by a significant impact on the anterior eye segment, including higher anterior and posterior keratometric values, lower anterior chamber depth, higher iris thickness, and narrower angle parameters. The characteristic anterior eye segment features of PEX syndrome can be detected using SS-OCT, which could potentially assist clinicians in properly managing the disease.

## 1. Introduction

Optical coherence tomography (OCT), is a non-contact precise tissue imaging technique that was developed in 1991 [1]. It has been broadly utilized for the diagnosis of conditions in the anterior segments and posterior segments of the eye, as well as for intra operative evaluations [2]. Several optical coherence tomography techniques are available, including Fourier-domain OCT (FD-OCT), spectral domain, time domain OCT, and OCT using swept source lasers (swept source OCT (SS-OCT)) [3,4,5]. The CASIA2 (Corporation Tomey, Nagoya, Japan) is a commercially available device with a longitudinal resolution of 10 μm, a scanning speed of 50,000 A-scans/s, and a transverse resolution of 30 μm. It is characterized by improved optical coherence, high reproducibility, and a scan frame size of 13 mm × 16 mm, which allows for data to be obtained from the surface of the cornea to the posterior surface of the lens in a single scan [6,7].

Pseudoexfoliation syndrome (PEX) is a systemic, age-related disorder characterized by the production and accumulation of pseudoexfoliation material. In cases of this syndrome, extra ocular deposits have been localized to the connective tissue portions—known as the septa—traversing the organ tissue; this is associated with elastic fibers, collagen fibers, fibroblasts, and the walls of small blood vessels, suggesting the systemic nature of PEX [8]. PEX is one of the most common causes of secondary open-angle glaucoma [9]. The genetic basis of PEX syndrome is steadily being elucidated [10]. As a result of well-powered genome-wide association studies and sequencing efforts, seven loci have been identified in association with the risk of PEX that surpass genome-wide significance. It has been proposed that the pericellular accumulation of pseudoexfoliation material may disrupt the normal cellular basement membrane, thereby leading to endothelial dysfunction [11]. Iris vasculopathy, endothelial basement membrane abnormalities, and lumen obliteration have been described in patients with PEX [12,13]. In light of the high risk of glaucoma development and potential complications during cataract surgery, an accurate and early diagnosis of PEX syndrome is of considerable clinical relevance [14]. Furthermore, PEX syndrome causes changes in the cornea. As such, eyes with PEX present decreased corneal sub-basal nerve plexus variables and cell densities in the cornea [15]. Patients with PEX were found to have decreased keratocyte stromal cell and basal corneal epithelial cell counts, in addition to sub-basal neural integrity [16,17].

This study was designed to identify the corneal thicknesses, differences between the anterior and posterior corneal surface parameters, elevation maps, Fourier indices, lens parameters, and iridocorneal parameters in patients with PEX and individuals with healthy eyes. To date, no detailed, complex accounts have been published on the corneal and iridocorneal angle characteristics in patients with pseudoexfoliation syndrome (PEX) using SS-OCT.

## 2. Materials and Methods

This study was performed at the Medical University of Silesia in Katowice, Department of Ophthalmology, Faculty of Medical Sciences in Zabrze. A total of 150 eyes of patients with pseudoexfoliation syndrome (ages 69–86 years) and 166 eyes in the control group (ages 54–79 years) were analyzed. Patients included in the control group were undergoing preventive screening for glaucoma. Only one eye was selected for each patient for anterior segment optical coherence tomography.

The study included patients whose slit-lamp examination revealed the presence of PEX syndrome without the presence of cataracts. Indicators included fibrillar white flaky deposits at the papillary border as well as on the anterior lens capsule. 

The exclusion criteria for both groups were as follows:

(I) Presence of anterior eye segment diseases (e.g., anterior uveitis, glaucoma, high myopia, or lens subluxation);
(II) Presence of corneal abnormalities affecting imaging (e.g., corneal opacity, corneal degeneration and dystrophies, or corneal scarring);
(III) History of eye surgery or injury;
(IV) Confirmation or suspicion of corneal ectasia;
(V) Poor fixation of the subject, leading to a low quality of the obtained images and a lack of cooperation with the researcher;
(VI) Contact lens wear.

Each participant was tested for both anterior and posterior probability of corneal ectasia (ESI-A and ESI-P) in both the control and PEX groups using the SS-OCT CASIA 2built-in software (Version 4C.4; Corporation Tomey, Nagoya, Japan). Both confirmation and suspicion of corneal ectasia (any measurement >0%) were adopted as part of the exclusion criteria for the study protocol. Eyes with refractive error higher than or equal to ±2.0 D were also excluded. According to the Lens Opacities Classification System III (LOCS III), cataract severity was measured with 10% phenylephrine hydrochloride and 1% tropicamid for 15 min in the dilated eye prior to examination [18]; a slit lamp was used at maximum illumination without light filtering for this measurement. This classification contained six degrees of the extent of nuclear color (NC1–NC6) and nuclear opacity (NO1–NO6). Only participants with a nuclear color of NC1 and nuclear opacity of NO1 were included in our study, as the presence of cataracts could have potentially interfered with our measurements, i.e., the depth of the anterior chamber. Additionally, patients with glaucoma (including PEX glaucoma) or those suspected of having glaucoma were excluded. Fundus examination was performed on each patient to assess the optic disc. An intraocular pressure test was also performed. If any abnormalities were detected, the patient subsequently underwent visual field examination and examination of the thickness of the nerve fibers of the retina. Any of the abnormalities listed above disqualified the patient from the study.

This controlled, prospective, observational study was conducted in accordance with the Declaration of Helsinki and was approved by the Bioethical Committee of the Medical University of Silesia in Katowice (KNW/0022/KB1/129/18). Imaging of the anterior segment of the eye was performed with CASIA2 (Corporation Tomey). All of the measurements were performed three times by the same operator during one visit and in the same sequence. All volunteers provided written consent to participate in the study.

During examinations, participants were asked to fix their eyesight on a marker. The examination was performed after establishing and aligning the optical axis of the eye with the device using the following protocols: “Lens Biometry”, “AS Global Scan”, and “Corneal Map”. Parameter measurements were automatically generated by the software embedded in the device. We used the “Lens Analysis” module in “Lens Biometry”, the “3DAnalysis” module in “AS Global Scan”, and the “Corneal Topography” module in “Corneal Map” to accomplish this task. The acceptance criteria for the images were as follows: clearly visible scleral spur, angle, and iris. The researchers divided the analyzed parameters of the anterior segment into three main groups based on the parameters describing the cornea, lens, and iridocorneal angle. The following parameters were analyzed: keratometry steep (Ks), keratometry flat (Kf), posterior keratometry steep (pKs), posterior keratometry flat (pKf), central corneal thickness (CCT), Fourier index spherical (Fi3(6)-Sph), Fourier index regular (Fi3(6)-Reg), Fourier index asymmetry (Fi3(6)-Asy), Fourier index higher order(Fi3-HO), TILT, decentralization (DECENT), anterior chamber depth (ACD), anterior chamber volume (ACV), angle opening distance (AOD 250,500,750), angle recess area (ARA 250,500,750), trabecular iris space area (TISA 250,500,750), and trabecular iris angle (TIA 250,500,750). The detailed descriptions of the parameters and the methodology were included in our previously published paper regarding anterior eye segment parameter analysis in normal subjects [19].

Statistical analysis was carried out using the Statistica (version 13.1.pl, StatSoft, Kraków, Poland) and the Panda and Pingouin packages in Python (StatSoft, Kraków, Poland). A *p*-value of <0.05 was considered significant. The normality of the data distribution was assessed using the Shapiro−Wilk test. Significant between-group differences were analyzed using the Student’s *t*-test for normally distributed data and the Mann−Whitney *U* test for non-normally distributed data. The correlations between the parameters were examined using Spearman correlation coefficients. The Mann−Whitney *U* test was used to compare the data by gender. The ANOVA and Kruskal−Wallis tests were used to compare data by age.

## 3. Results

### 3.1. Demographic Data

A total of 150 eyes of patients with pseudoexfoliation syndrome (ages 69–86 years) and 166 eyes in the control group (ages 54–79 years) were analyzed. Selected groups were sex and age, and were intraocular pressure matched. Detailed demographic data characteristics are included in Table 1. The mean intraocular pressure was 15.41 ± 2.82 (range: 11–20) mmHg in the PEX group and 14.67 ± 3.02 (range: 11–19) mmHg in the control group.

There were no statistically significant differences in intraocular pressure between the study groups (*p* = 0.23; Mann−Whitney *U* test). The study included 102 (68%) women and 48 (32%) men in the PEX group, and 92 (55.4%) women and 74 (44.6%) men in the control group. There were more women than men in the PEX group (*p* = 0.07).

The mean age of the patients in the PEX group was 74.76 years (SD ± 4.736) and 66.162 years (SD ± 7.523) in the control group. Patients in the control group were slightly younger than in the PEX group, but the difference remained statistically insignificant (*p* = 0.14).

The results of the data analysis in terms of age and sex in the healthy subjects (control group) are included in our previous work, “Optical Coherence Tomography Analysis of the Selected Eye’s Anterior Segment Parameters” [19]. 

The patients in the PEX group were divided into three age groups: 60–70, 70–80, and >80 years old. The patients were further divided into two groups depending on gender: women and men.

### 3.2. OCT Parameters of the Cornea

The central corneal thickness in the PXS group was 549.56 µm and was slightly larger than that of the control group (540.56 µm). The difference was not statistically significant (*p* = 0.63; Mann−Whitney *U* test). We observed statistically significant differences in both groups regarding the keratometric values. Both the Ks (43.13 Din the control group vs. 44.68 D in the PEX group) and Kf (42.10 D vs. 43.59 D) values differed significantly between the two groups (*p* < 0.01). The same trend was observed for the posterior corneal curvature, as both pKs (−6.18 D vs.−6.35 D) and pKf (−5.84 D vs. −6.03 D) were significantly different between the two groups (*p* < 0.01). The parameters of the cornea are summarized in Table 2. With regard to sex and age, no statistical significance was found for the following cornea parameters: Ks, Kf, Ks p, Kf p, and CCT. The Mann−Whitney *U* test was used to compare the data by gender. The ANOVA and Kruskal−Wallis tests were used to compare data depending on age, as seen in Table S1 (Table S1 is available in the Supplementary Materials).

### 3.3. Lens Analysis

The mean (±SD) TILT (tilt of the lens axis in relation to the vision axis) in the PXS group was 5.22° (±1.67). The mean (±SD) TILT in the control group was 5.04° (±1.26). The TILT parameter was normally distributed only in the control group (*p* = 0.14 in the control group and *p* = 0.01 in the PXS group for the Shapiro−Wilk test). In the PXS group, the mean (±SD) DECENT (the decentralization of the lens in relation to the visual axis) was 0.202 mm (±0.08). In the control group, this value was practically identical at 0.202 mm (±0.09). This parameter had a normal distribution only in the PXS group (*p* = 0.37 in the PXS group and *p* = 0.001 in the control group). Statistically significant differences between the studied and control groups were not observed for either TILT or DECENT (*p* = 0.53 and *p* = 0.89; Mann−Whitney *U* test). No statistical significance was found for the following lens parameters: TILT and DECENT (ANOVA and Kruskal−Wallis and Mann−Whitney *U* tests). The parameters of the lens of the eye are summarized in Table 3 and Table S1.

### 3.4. Fourier Analysis of the Cornea and Anterior Chamber Depth

The anterior chamber of the eye was visibly shallower in patients with PEX syndrome than in those of the control group (2.49 mm vs. 3.07 mm; *p* < 0.001). In fact, the keratometric Fourier analysis parameters of the cornea showed many differences between the PEX and control groups. Fi-3-Sph and Fi-6-Sph were significantly different between both groups (*p* < 0.001; Mann−Whitney *U* test). Fi-3-Ass, Fi-3-HO, Fi-6-Ass, and Fi-6-HO were also significantly different between the two groups (for Fi-3-Ass, Fi-3-HO, and Fi-6-Ass, *p* < 0.01; Fi-6-HO *p* < 0.001; Mann−Whitney *U* test). Among all of the parameters, only Fi-3-Reg and Fi-6-Reg demonstrated no significant differences between the two groups (*p* = 0.19, *p* = 0.12). No statistical differences were detected for the Fourier index parameters in the PEX group with regard to sex and age (ANOVA, Kruskal−Wallis, and Mann−Whitney *U* tests). The results are summarized in Table 4 and Table S1.

### 3.5. Iris Parameters

Regarding the iris area parameters, statistically significant differences were present between the PEX and control groups in all four quadrants of the eye (180-I-Area and 270-i-Area (*p* < 0.001; Student’s *t*-test) and 0-I-Area and 90-I-Area (*p* < 0.001; Mann−Whitney *U* test)). The I-area parameter was generally greater in the PXS group. This corresponded to the thicker irises found in the eyes with PXS. 

The iris curvature also differed between the two groups. Specifically, all four parameters—namely 0-I-Curv, 90-I-Curv, 180-I-Curv, and 270-I-Curv—were statistically different (*p* < 0.001; Mann−Whitney *U* test). Concave, flat, and convex-shaped irises were found in the eyes within the control group. However, no concave-shaped irises were found in the PXS eyes at all. 

### 3.6. Angle Parameters

**Figure 1:**
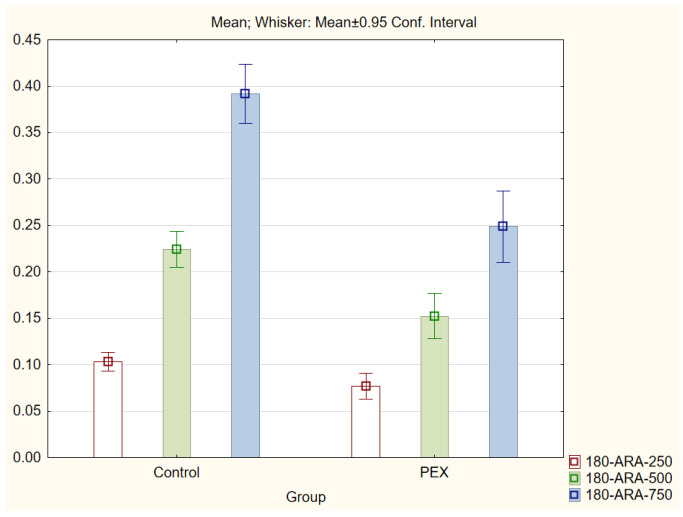
Comparison of the results of the angle recess area (ARA 250, 500, and 750) in the PEX group and the control group.

**Figure 2:**
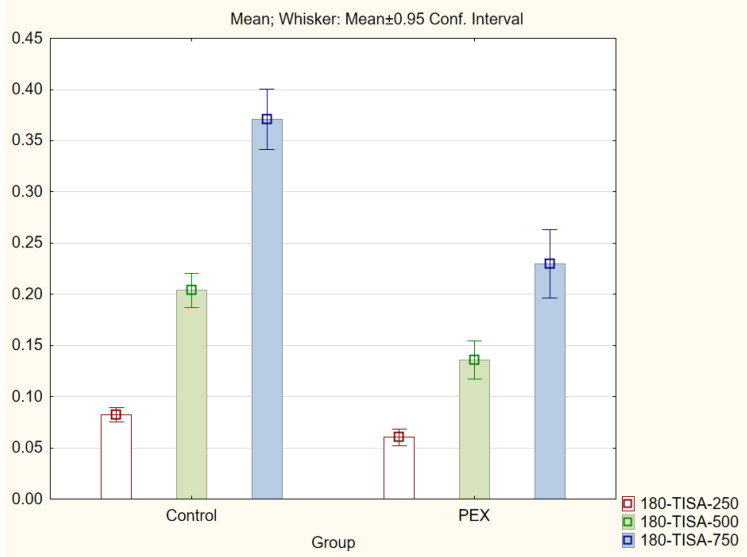
Comparison of the results of the trabecular iris space area (TISA 250, 500, and 750) in the PEX group and the control group.

**Figure 3:**
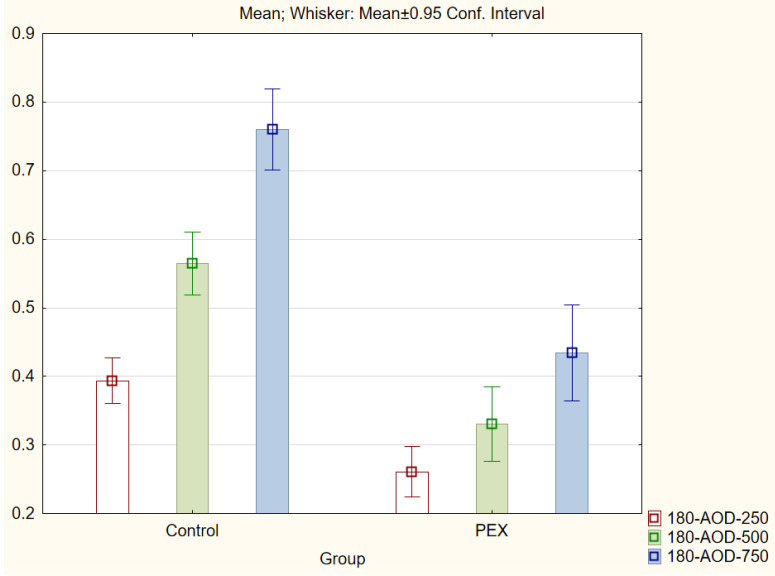
Comparison of the results of the angle opening distance (AOD 250, 500, and 750) in the PEX group and the control group.

**Figure 4:**
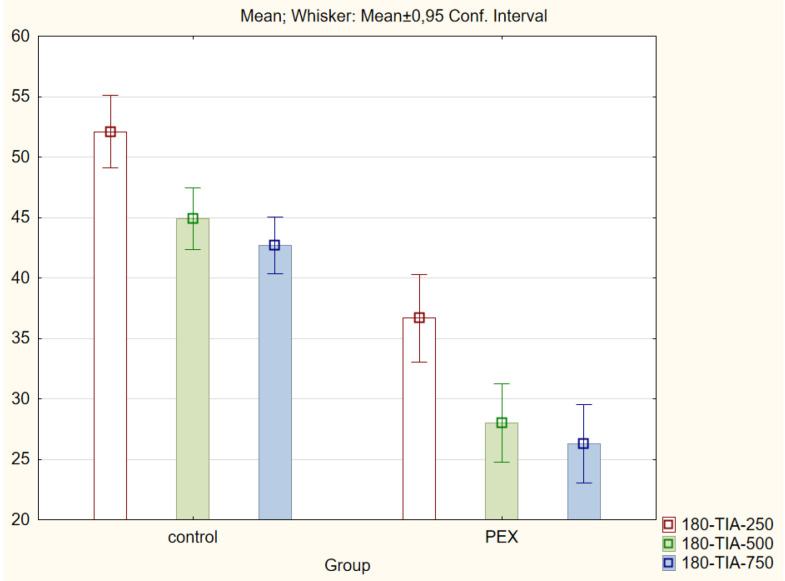
Comparison of the results of the trabecular iris angle (TIA 250, 500, and 750) in the PEX group and the control group.

With regard to the trabecular iris angle (TIA) parameter, statistically significant differences were found in all four quadrants of the eye at distances of 750 μm and 500 μm (*p* < 0.001). The 0-TIA-250 parameter was the only parameter that did not display differences between the two groups. The ARA, AOD, and TISA parameters were also lower in the PEX group (*p* < 0.01; Mann−Whitney *U* test) for all quadrants and distances (Figure 1, Figure 2, Figure 3 and Figure 4).

**Figure 5:**
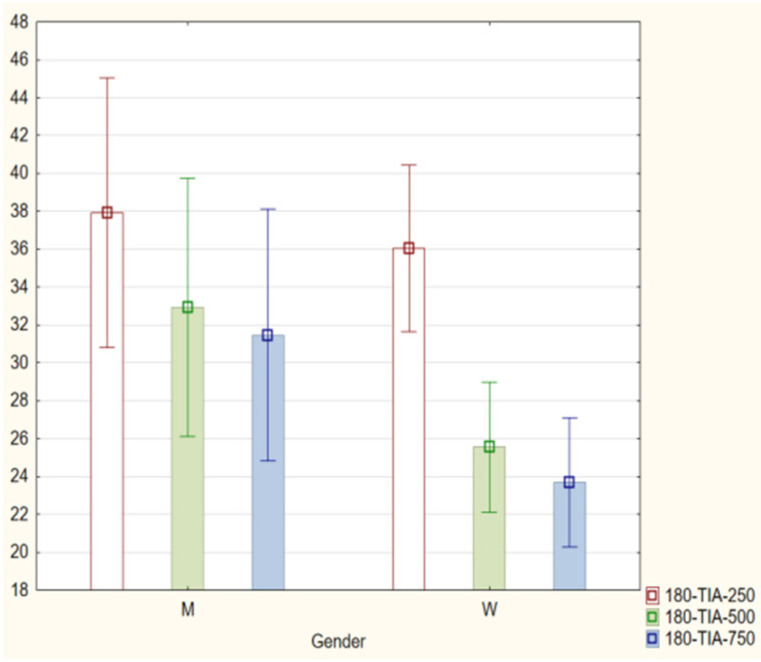
Comparison of the results of the angle parameters based on age.

With regard to sex and age, no statistical significance was found in the PEX group for the following iridocorneal angle parameters: ARA, TISA, AOD, are TIA in all quadrants (0°, 90°, 180°, and 270°). Data are available in Table S2 of Supplementary Materials. The Mann−Whitney *U* test was used to compare the data by gender. The ANOVA and Kruskal−Wallis tests were used to compare data by age (Figure 5).

Additional diagrams are available in the Supplementary Materials as Figures S1–S3.

## 4. Discussion

Pseudoexfoliation syndrome (PEX) is a complex disorder in terms of its influence on the anterior chamber structures of the eye. PEX material is found on the corneal stroma, corneal epithelial basement membrane, corneal endothelial cells, lens capsule, lens zonules, trabecular meshwork, pupillary border, and iris [20]. In this study, we focused on defining the detailed characteristic features of the corneal, iris, and angle parameters, as well as the Fourier indices based on the SS-OCT of normal and PEX eye patients. AS-OCT has revolutionized anterior segment imaging. However, studies on AS-OCT in PEX remain scarce.

In terms of central corneal thickness, our study demonstrated no statistically significant differences between the PEX and control groups. These results are in line with those of the majority of the papers found in the extant literature. We analyzed studies regarding CCT in the eyes of patients with PEX syndrome, and eight of these studies also found no statistically significant differences with regard to CCT. The following studies were included: Kaljurand et al. (2007; 520 vs. 515 µm; control vs. PEX) [21], Ostern et al. (2012; 550 vs. 554) [22]; de Juan-Marcos et al. (2013; 540 vs. 531) [23], Hayashi et al. (2013; 530 vs. 533) [24], Tomaszewski et al. (2014; 527 vs. 529) [25], Demircan et al. (2015; 508 vs. 499) [26], Kocabeyoglu et al. (2016; 541 vs. 537) [27], and Sarowa et al. (2016; 530 vs. 525) [28].

At the same time, CCT differed significantly between groups in three studies: Puska et al. (2000, 523 vs. 528) [29], Inoue et al. (2003, 547 vs. 528) [30], and Yuksel et al. (2016, 558 vs. 543) [31]. The variation in these results could be explained by the differences in the characteristics of the studied populations and measurement devices.

The presence of PEX is a critical point in terms of cataract surgery. Many studies have found a statistically significant increase in intraoperative and postoperative complications concerning cataract extraction in eyes with PEX [32,33,34]. Zonular dialysis can occur in up to 15% of PEX eyes undergoing cataract surgery [34]. In a study on 1052 patients, Scorolli et al. found that eyes with PEX have a five times greater risk of intraoperative complications in cataract surgery compared to normal cases. This particular study emphasized that the recognition of this condition is vital, particularly prior to performing surgery on such patients [33]. In our study, the lens parameters that could be connected with preoperative lens subluxation remained nearly identical between the PEX and control groups. As we excluded four patients (2.4% total) with lens subluxation, this result coincides with our expectations. We found no papers in the literature discussing Fourier analysis of the PEX cornea. Fourier analysis parameters (keratometric) showed especially greater higher order irregularities and asymmetric refractive power of the PEX corneas. These findings occurred despite the lack of variation in CCT between the groups. PEX can lead to corneal endothelial cell decompensation; this can result in severe keratopathy, which requires keratoplasty [35]. As pseudoexfoliative material accumulates in many eye structures, including all corneal layers [16], these observations may reflect changes in the corneal microstructure. Thus, further investigations should be conducted regarding this matter. 

In our study, iris thickness was greater in the PEX group. Various authors also investigated iris morphology using AS-OCT in PEX eyes. For instance, Rao et al. found the iris volume to be greater in the eyes with PXS than those with primary open glaucoma. Notably, in the same study, iris thickness showed no differences between groups. However, the authors did not compare eyes with PEX to normal eyes, as the study aim was to determine the predictors of glaucoma in eyes with PEX and not the detection of PEX using AS-OCT [36].

Our study demonstrated that the AS-OCT-measured angle parameters in the PEX group were significantly smaller compared to those that were considered normal. This was in agreement with the gonioscopic evaluation, which showed that up to 32% of patients with PEX had narrow angles compared to the 4–10% present in the general population [37,38].

The anterior chamber was significantly shallower in PEX eyes compared to the normal ones. This finding was also reported by Zheng et al. in their study based on an AS-OCT device [39]. In 2021, Mohammadi et al. compared normal and PEX eyes using AS-OCT CASIA [40]. In the study, the ACD was smaller in eyes with PEX and PEXG compared to the eyes in the control group; no difference was found between the eyes with PEX and PEXG. However, a progressive decrease was present in the angle parameters from the eyes of the controls to those of the PEX and to those of the PEXG. We agree with the authors in their conclusion; the anterior shift of the lens—secondary to the subclinical weakness of zonular fibers—plays a key role in the narrowing of the anterior chamber angle in cases of PEX. Our study also confirmed that a thick iris, combined with a smaller ACD and lower angle parameters, may play a role in the development of PEX glaucoma in PEX patients. These parameters may also help to distinguish patients with a higher risk of developing PEX glaucoma in the future. However, this hypothesis requires further investigation.

Our study demonstrated that the AS-OCT-measured angle, corneal, and lens parameters in the PEX group were not significantly different with regard to age and gender. There were no statistically significant differences in intraocular pressure between the study groups. No comparison of the demographic data has been published regarding age and sex, and their effect on the parameters of the anterior segment in the PEX group in the optical coherence tomography of the anterior segment. The eyes in the PEX group were of a narrow age range, and this is likely the reason that no age-related differences were detected.

This study is not without certain limitations. The relatively small sample size could potentially have affected the study power. Furthermore, we did not enroll patients with PEX glaucoma in the study. Therefore, the causal relationship was not determined between AS-OCT parameter changes and PEX progression to glaucoma. As such, we will make an effort to increase the study group size and enroll patients with glaucomatous changes in future studies for further analysis. Additionally, the impact of other effects—such as the disturbance of the tear film, internal/indoor environmental factors, the time of day, or the presence of other ocular factors—cannot be ruled out. It should also be taken into account that PEX may not be the only factor influencing the measurements. Finally, the study was conducted on Polish citizens. Therefore, the results may not be applicable to other racial or ethnic groups.

## 5. Conclusions

PEX syndrome is characterized by its significant impact on the anterior eye segment, which includes higher anterior and posterior keratometric values, lower anterior chamber depth, increased iris thickness, and narrower angle parameters. With regard to gender and age, no statistically significant differences were found among the PEX group for the iridocorneal angle parameters, corneal parameters, and lens parameters. Characteristic anterior eye segment features of PEX syndrome can be detected by anterior segment OCT, which could potentially assist clinicians in properly managing the disease.

## Figures and Tables

**Table 1:** Demographic data.

	Total	PEX Group	Control Group	p*
**Age**				*p* = 0.14
Mean ± SD	69.32 ± 7.813	74.76 ± 4.736	66.162 ± 7.523	
Median	71	74	65	
Range	54–86	69–86	54–79	
**Sex**	Women:194	102 (68%)	92 (55.4%)	*p* = 0.07
	Men: 122	48 (32%)	74 (44.6%)	
**Intraocular pressure**		15.41 (SD ± 2.82) mmHg Range: 11–20 mmHg	14.67 (SD ± 3.02) mmHg Range: 11–19 mmHg	*p* = 0.23

p*: *p* for the significance of differences between the control and study groups.

**Table 2:** Comparison of the cornea parameters in the PEX group and in the control group.

	Control Group						PEX						p*
Parameter	Mean	STD	IC95	Median	Minimum	Maximum	Mean	STD	IC95	Median	Minimum	Maximum	
Ks	43.13	4.77	44.16	43.80	1.27	46.50	44.68	2.00	2.50	44.80	40.30	49.20	**<0.001**
Kf	42.10	4.66	43.11	42.55	1.22	46.10	43.59	1.75	2.19	43.70	39.70	47.90	**<0.001**
Ks p	−6.18	0.73	−6.02	−6.30	−7.00	0.21	−6.35	0.32	0.40	−6.40	−6.90	−5.70	0.02
Kf p	−5.84	0.69	−5.69	−5.90	−6.50	0.20	−6.03	0.29	0.37	−6.10	−6.50	−5.400	**0.008**
CCT	540.56	62.91	554.13	549.00	28.82	618.00	549.56	33.58	41.85	542.0	486.00	623.00	0.63

p*: *p* for the significance of differences between the control and study groups.

**Table 3:** Comparison of the results of the lens analysis.

	Control Group						PEX Group						p*
Parameter	Mean	STD	IC95	Median	Minimum	Maximum	Mean	STD	IC95	Median	Minimum	Maximum	
TILT	5.04	1.26	5.31	4.85	1.19	9.00	5.22	1.67	2.11	5.10	1.50	11.50	0.53
DECENT	0.20	0.09	0.22	0.18	0.02	0.58	0.20	0.08	0.11	0.19	0.01	0.42	0.89

p*: *p* for the significance of differences between the control and study groups.

**Table 4:** Comparison of the results of the Fourier analysis of the cornea and anterior chamber depth.

	Control Group						PEX Group						p*
Parameter	Mean	STD	IC95	Median	Minimum	Maximum	Mean	STD	IC95	Median	Minimum	Maximum	
ACD	3.0728	0.43130	3.1659	3.0900	0.31167	3.8700	2.4941	0.40721	0.5087	2.4700	1.6000	3.4900	**<0.001**
FI-3-Sph	42.6011	4.71227	43.6175	43.2400	1.22978	46.3600	44.0596	1.79889	2.2473	44.1700	39.9700	48.5400	**<0.001**
FI-3-Reg	0.4938	0.24573	0.5468	0.4800	0.02000	1.4400	0.5257	0.48367	0.6042	0.3900	0.0400	2.7100	0.19
FI-3-Ass	0.2386	0.12395	0.2654	0.2200	0.03000	0.7300	0.3482	0.25557	0.3193	0.2800	0.0300	1.3300	**<0.01**
FI-3-HO	0.1532	0.05408	0.1649	0.1500	0.05059	0.3200	0.1802	0.07712	0.0963	0.1700	0.0900	0.5700	**<0.001**
FI-6-Sph	42.4245	4.68880	43.4358	43.0800	1.21124	45.9200	43.9478	1.77357	2.2157	43.9400	39.8700	48.3900	**<0.01**
FI-6-Reg	0.5027	0.24011	0.5545	0.4700	0.14000	1.4100	0.5200	0.46182	0.5769	0.3800	0.0900	2.5400	0.12
FI-6-Ass	0.3154	0.15791	0.3494	0.3100	0.07000	0.9300	0.4384	0.29779	0.3720	0.3400	0.0700	1.5500	0.01
FI-6-HO	0.1758	0.07147	0.1913	0.1600	0.07071	0.6300	0.2092	0.07976	0.0996	0.1900	0.1100	0.6600	**<0.001**

p*: *p* for the significance of differences between the control and study groups.

## Data Availability

The data presented in this study are available in Chair and Clinical Department of Ophthalmology, Faculty of Medical Sciences in Zabrze, Medical University of Silesia in Katowice, 40-055 Katowice, Poland.

## Supplementary Materials

The following supporting information can be downloaded at: https://www.mdpi.com/article/10.3390/jcm11010268/s1, Figure S1: Gender comparison for idirocorneal parameters; Figure S2: Age comparison for TIA (ANOVA Kruskal Wallis, *p* < 0.05); Figure S3: Age comparison for iridocorneal parameters; Table S1: Gender comparison in the PEX group for corneal, Fourier and lens parameters (Mann Whitney *U* test); Table S2: Gender comparison in the PEX group for the iridocorneal parameters (Mann Whitney *U* test).
