# Supplementary material for: Swept Source Optical Coherence Tomography Analysis of a Selected Eye’s Anterior Segment Parameters in Patients with Pseudoexfoliation Syndrome
Source: J Clin Med. 2022 Jan 5;11(1):268. doi: 10.3390/jcm11010268 (PMC8745779; doi:10.3390/jcm11010268)
Supplement: Supplementary file 1 [file jcm-11-00268-s001.zip › jcm-1522245-supplementary.pdf]

Table S1. Gender comparison in the PEX group for corneal. Fourier and lens parameters (Mann Whitney U test).

TILT	341.5000	693.5000	197.5000	0.465822	0.641343	0.466007	0.641211	0.636491
DECENT	304.5000	730.5000	199.5000	-0.416789	0.676833	-0.417160	0.676562	0.671520
Ks	374.5000	850.5000	238.5000	-0.533002	0.594033	-0.533410	0.593750	0.590392
Kf	389.0000	836.0000	253.0000	-0.223861	0.822866	-0.223969	0.822781	0.824745
Ks p	435.5000	789.5000	228.5000	0.746203	0.455546	0.749923	0.453302	0.453195
Kf p	418.0000	807.0000	246.0000	0.373101	0.709073	0.375776	0.707084	0.711983
CCT	398.5000	876.5000	262.5000	-0.187175	0.851523	-0.187270	0.851449	0.845041
ACD	491.5000	733.5000	172.5000	1.940127	0.052365	1.940523	0.052317	0.050366
FI-3-Sph	382.5000	842.5000	246.5000	-0.362441	0.717023	-0.362469	0.717002	0.711983
FI-3-Reg	417.0000	808.0000	247.0000	0.351781	0.725003	0.351889	0.724922	0.727781
FI-3-Ass	449.5000	775.5000	214.5000	1.044684	0.296170	1.045484	0.295800	0.294658
FI-3-HO	383.0000	842.0000	247.0000	-0.351781	0.725003	-0.353008	0.724083	0.727781
FI-6-Sph	384.5000	840.5000	248.5000	-0.319801	0.749119	-0.319817	0.749107	0.743694
FI-6-Reg	424.0000	801.0000	240.0000	0.501022	0.616356	0.501239	0.616203	0.619914
FI-6-Ass	415.0000	810.0000	249.0000	0.309141	0.757214	0.309275	0.757112	0.759714
FI-6-HO	362.0000	863.0000	226.0000	-0.799503	0.424000	-0.803903	0.421453	0.428042

Table S2. Gender comparison in the PEX group for the iridocorneal parameters (Mann Whitney U test).

	Rank Sum M		U	Z	p	Rank Sum M	p	2 side p
180-AOD-250	362.0000	814.0000	226.0000	-0.645178	0.518812	-0.645318	0.518721	0.522818
180-AOD-500	475.5000	700.5000	172.5000	1.815247	0.069487	1.815345	0.069472	0.067469
180-AOD-750	474.0000	702.0000	174.0000	1.782441	0.074678	1.782490	0.074670	0.074480
180-ARA-250	355.5000	820.5000	219.5000	-0.787336	0.431086	-0.787678	0.430886	0.428787
180-ARA-500	406.0000	770.0000	242.0000	0.295251	0.767802	0.295315	0.767753	0.770224
180-ARA-750	444.5000	731.5000	203.5000	1.137263	0.255429	1.137510	0.255326	0.253886
180-TISA-250	367.0000	809.0000	231.0000	-0.535826	0.592079	-0.536452	0.591646	0.595797
180-TISA-500	411.0000	765.0000	237.0000	0.404603	0.685769	0.404647	0.685737	0.688884
180-TISA-750	456.5000	719.5000	191.5000	1.399708	0.161602	1.399822	0.161567	0.159947
180-TIA-250	389.0000	787.0000	253.0000	-0.054676	0.956397	-0.054685	0.956389	0.956877
180-TIA-500	476.5000	699.5000	171.5000	1.837117	0.066193	1.837217	0.066179	0.064168
180-TIA-750	481.5000	694.5000	166.5000	1.946470	0.051599	1.946681	0.051574	0.049562

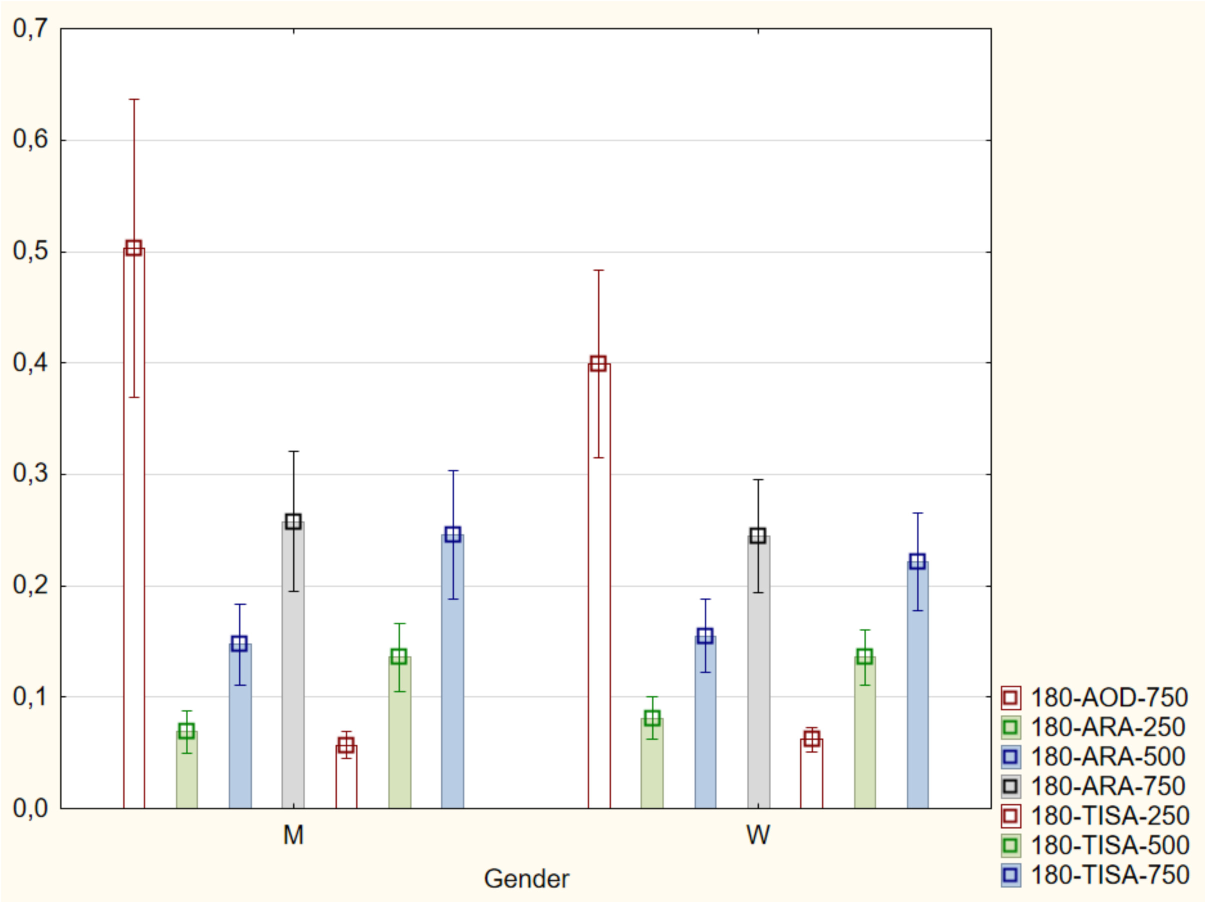

Fig. S1 Gender comparison for idirocorneal parameters.

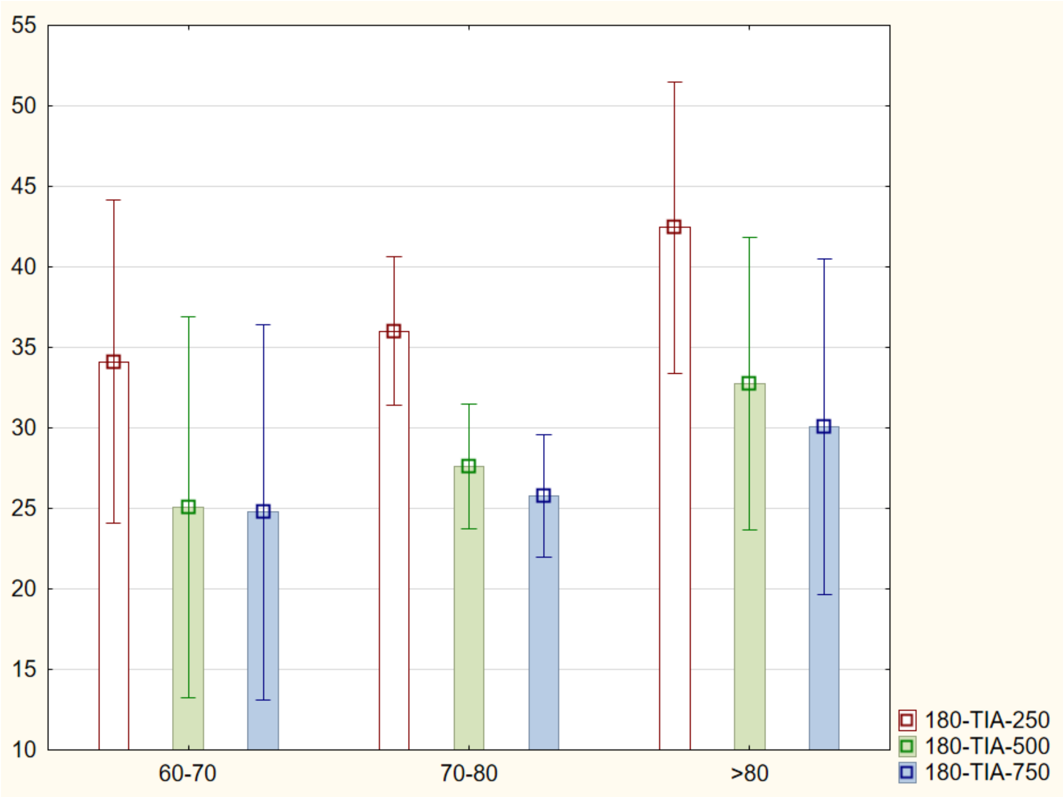

Figure S2. Age comparison for TIA (ANOVA Kruskal Wallis. $p < 0.05$)

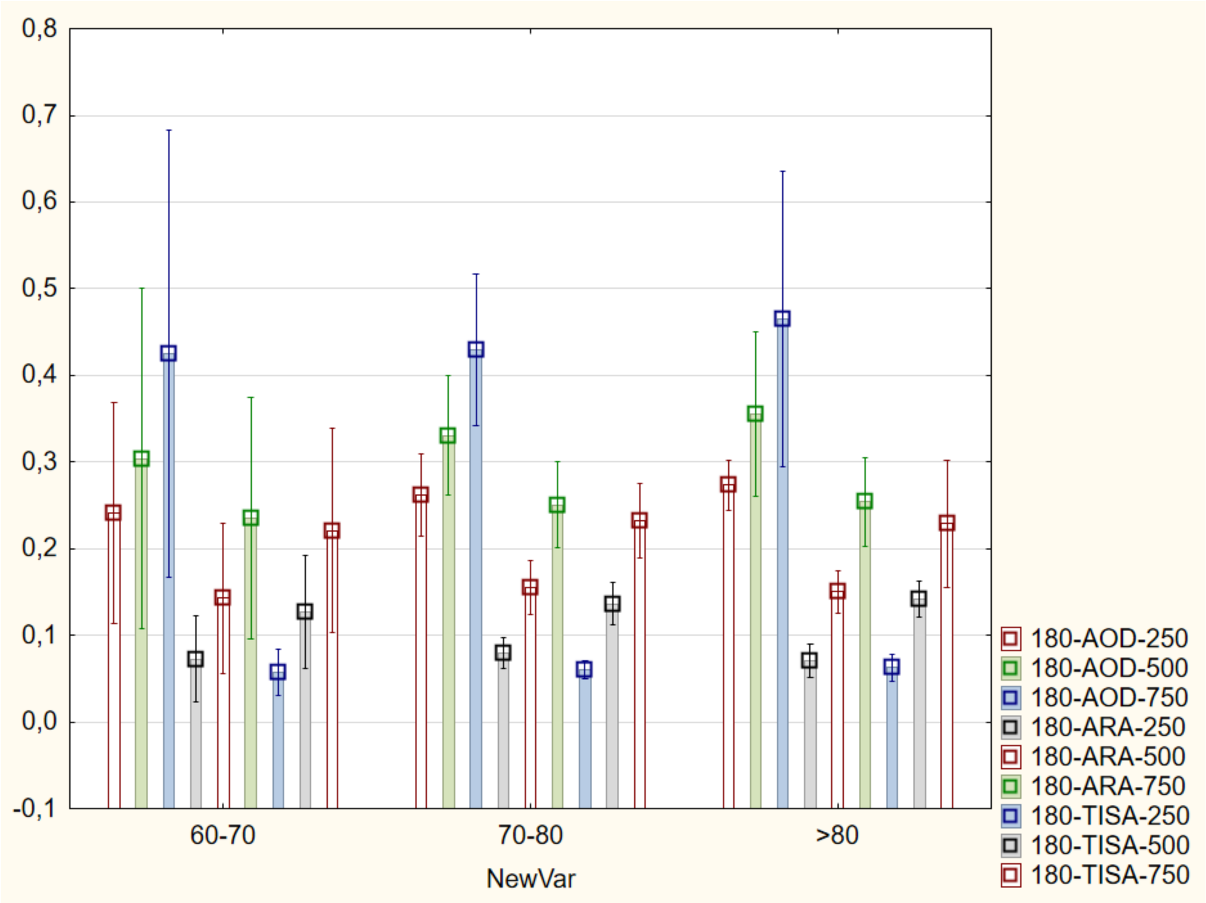

Figure S3. Age comparison for iridocorneal parameters.
